# Supplementary material for: KDM3B inhibitors disrupt the oncogenic activity of PAX3-FOXO1 in fusion-positive rhabdomyosarcoma
Source: Nat Commun. 2024 Feb 24;15:1703. doi: 10.1038/s41467-024-45902-y (PMC10894237; doi:10.1038/s41467-024-45902-y)
Supplement: Supplementary file 1 — Supplementary Information [file 41467_2024_45902_MOESM1_ESM.pdf]

SUPPLEMENTARY FIGURES

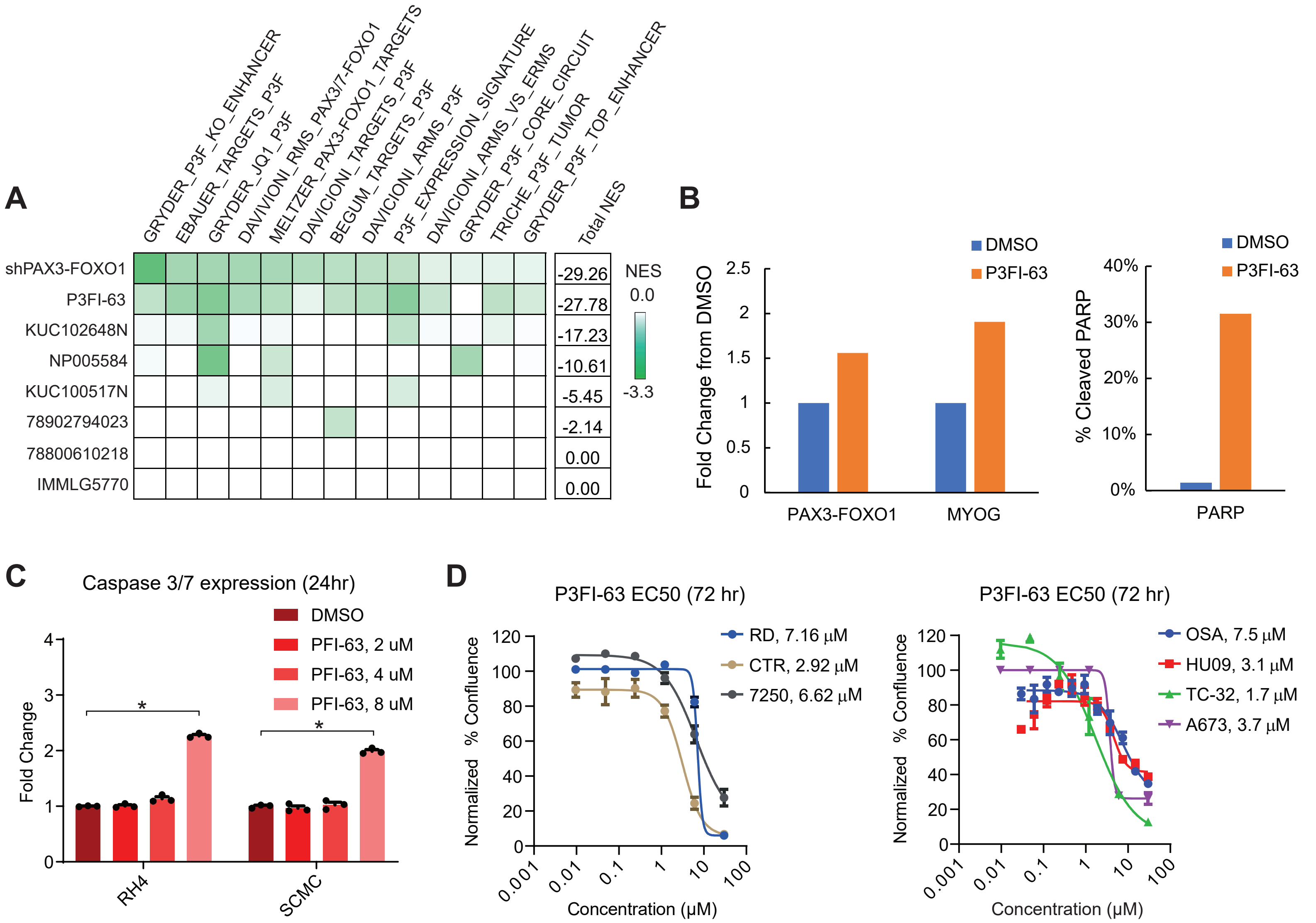

Supplementary Figure 1. A, RNA-seq GSEA analysis of top 7 uncharacterized novel compounds on PAX3-FOXO1 gene sets. Normalized enrichment score (NES). n=1. **B,** Quantitation of Western bands normalized to loading control and fold change based on DMSO. n=1. **C,** Quantitation of caspase 3/7 activity following treatment with P3FI-63 using the luminescence-based assay Caspase-Glo. n=3 biological replicates. Data presented as mean values +/- SEM. RH4 * $p=1.0 \times 10^{-9}$, SCMC * $p=1.1 \times 10^{-7}$ by 1-way ANOVA corrected for multiple testing. **D,** EC50 of growth inhibition of fusion negative RMS, human fibroblast 7250, and other pediatric cancer cell lines treated with P3FI-63. n=3 biological replicates. Data presented as mean values +/- SEM. All error bars = Standard error. Source data are provided as a Source Data file.

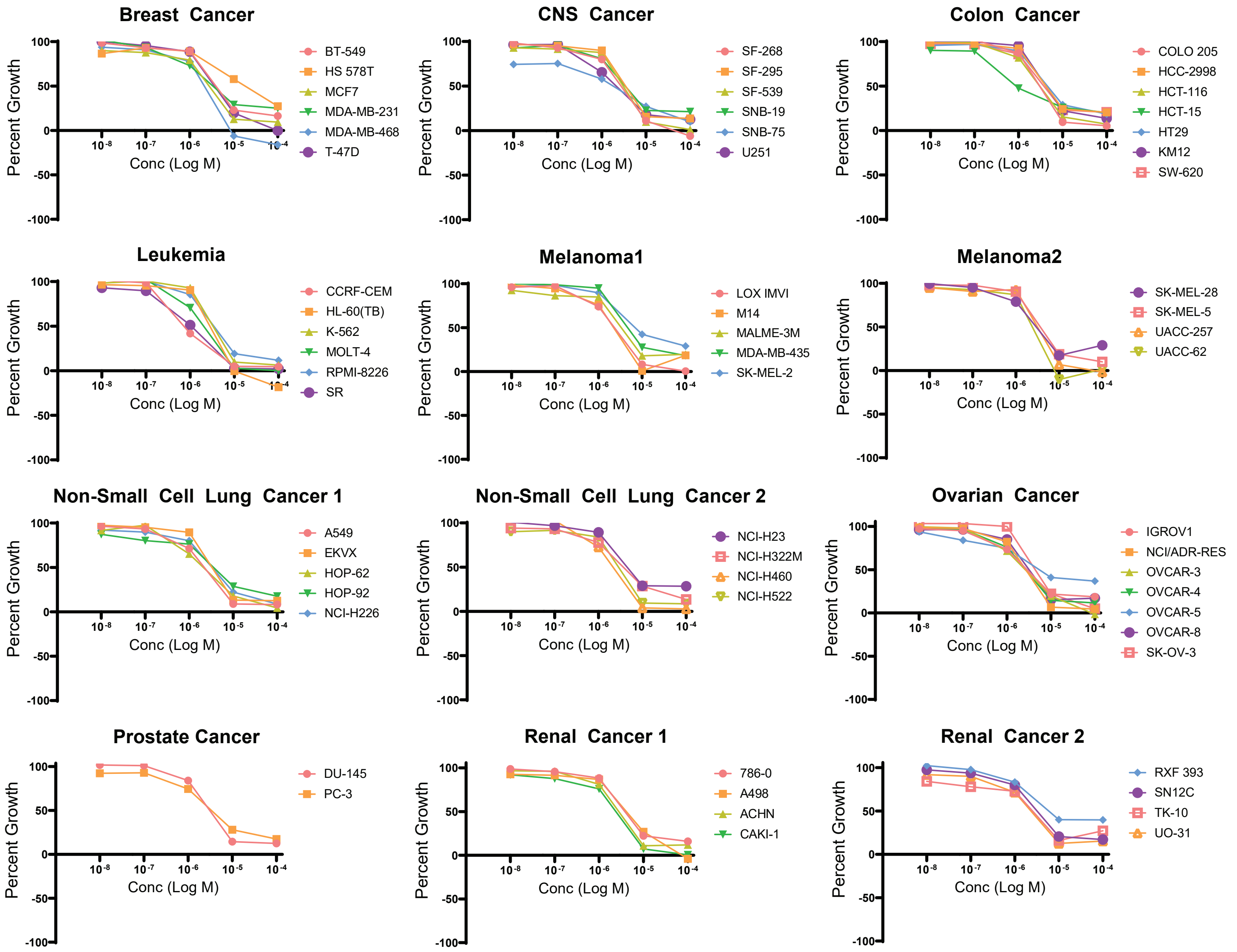

Supplementary Figure 2. Growth inhibition as percent of control in NCI-60 cancer cell lines treated with P3FI-63. n=2 biological replicates. Data presented as mean values. Source data are provided as a Source Data file.

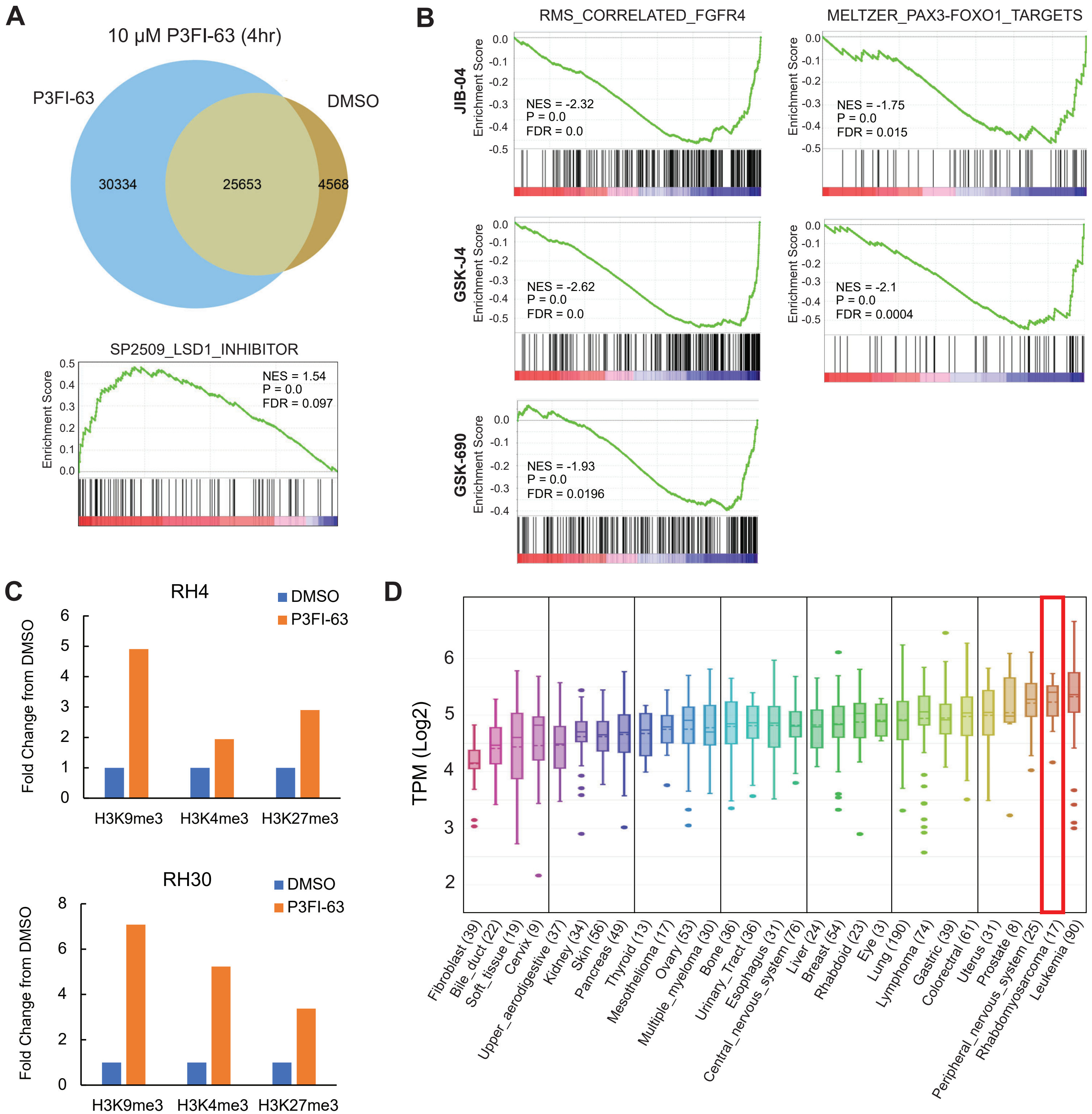

Supplementary Figure 3. A, ATAC-seq analysis after treatment with P3FI-63 vs DMSO for 4 hr. Venn diagram of ATAC-seq and GSEA analysis of P3FI-63 only genes. n=1. **B**, RNA-seq GSEA of KDM inhibitors JIB-04, GSK-J4, and GSK-690 impacting PAX3-FOXO1-associated gene sets. n=1 **C**, Quantitation of Western for histone marks. **D,** BROAD DepMap expression level of KDM3B in various cancer lines. Box plot with solid line being median and dotted line being mean. Box plots of quartiles with whiskers showing 1.5×interquartile ranges. Ordered according to mean.

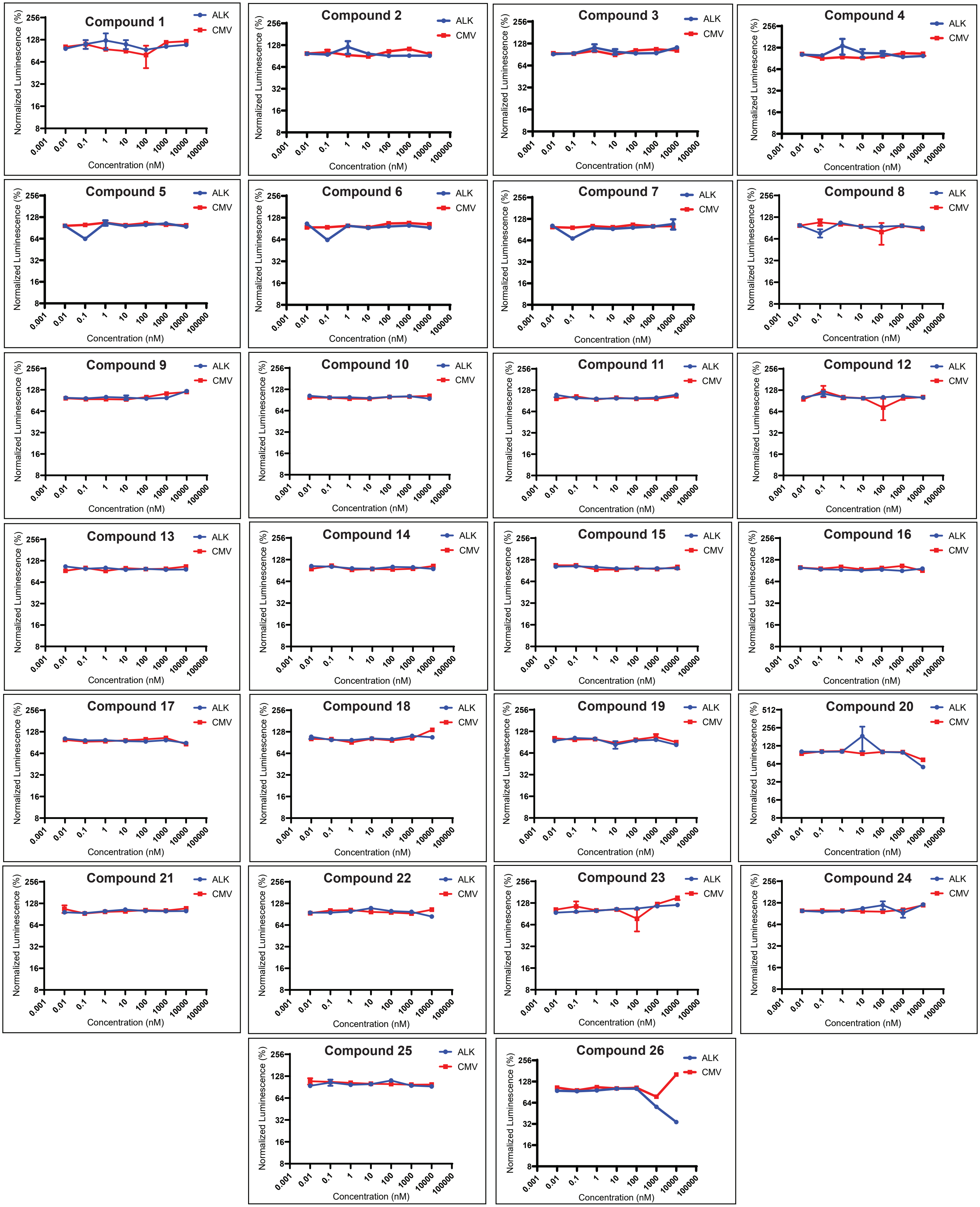

Supplementary Figure 4. Twenty-six analogs of P3FI-63 screen using *ALK*-Luc and CMV-Luc cell lines. n=4 biological replicates. Data presented as mean values +/- SEM. All error bars = Standard error.

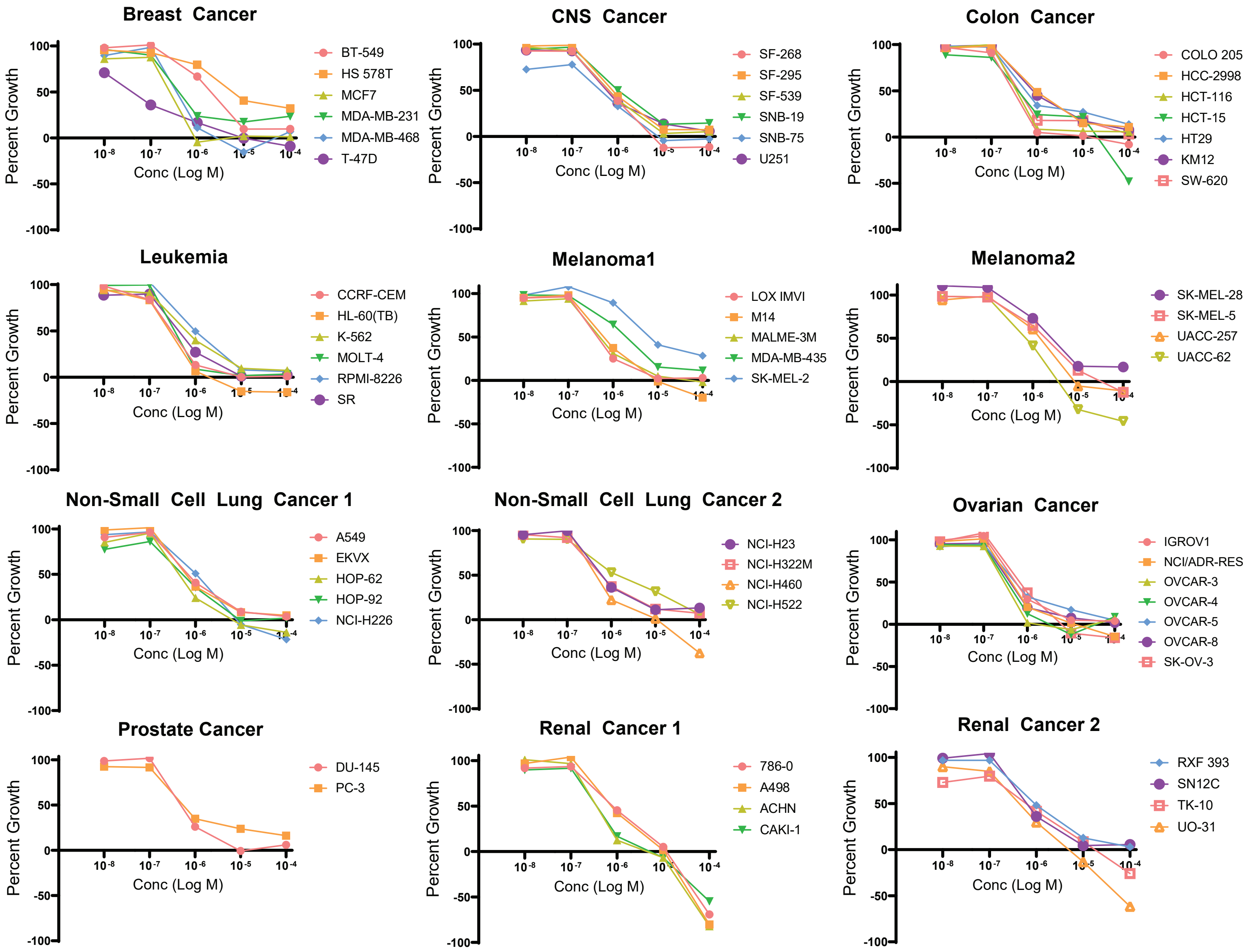

Supplementary Figure 5. Growth inhibition as percent of control in NCI-60 cancer cell lines treated with P3FI-90. n=2 biological replicates. Data presented as mean values. Source data are provided as a Source Data file.

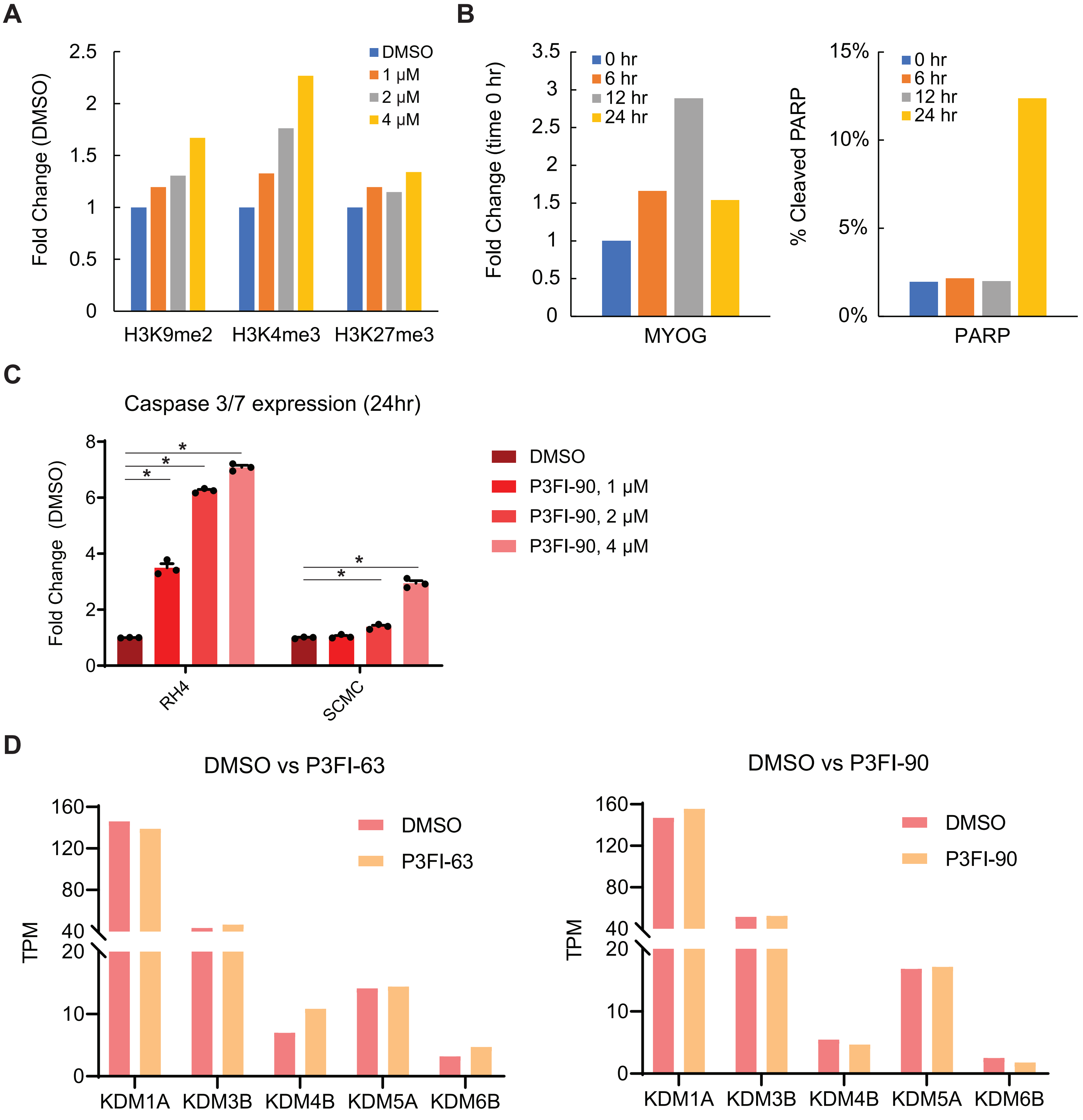

Supplementary Figure 6. A, Western blot quantitation of histone marks in RH4 cell line with DMSO control and various doses of P3FI-90. **B,** Western blot quantitation of MYOG and PARP cleavage as time course with P3FI-90 treatment at 1 μM. Fold change from time 0 to 6 hr, 12 hr, and 24 hr in RH4 cell line. **C,** Quantitation of caspase 3/7 activity using Caspase-Glo luminescence as read out. n=3 biological replicates. Data presented as mean values +/- SEM. RH4 1 μM $p=9.5x10^{-8}$, 2 μM $p=2.6x10^{-10}$, 4 μM $p=8.0x10^{-11}$. SCMC 2 μM p=0.0037, 4 μM $p=2.9x10^{-8}$ by 1-way ANOVA adjusted for multiple testing. Error bar = Standard error. **D,** RNA-seq transcript levels in transcripts per million (TPM) of KDMs in RH4 cell line after treatment with P3FI-63 (10μM) and P3FI-90 (1μM) vs DMSO. 6 hr time for P3FI-63 and 24 hr time point for P3FI-90. Source data are provided as a Source Data file.

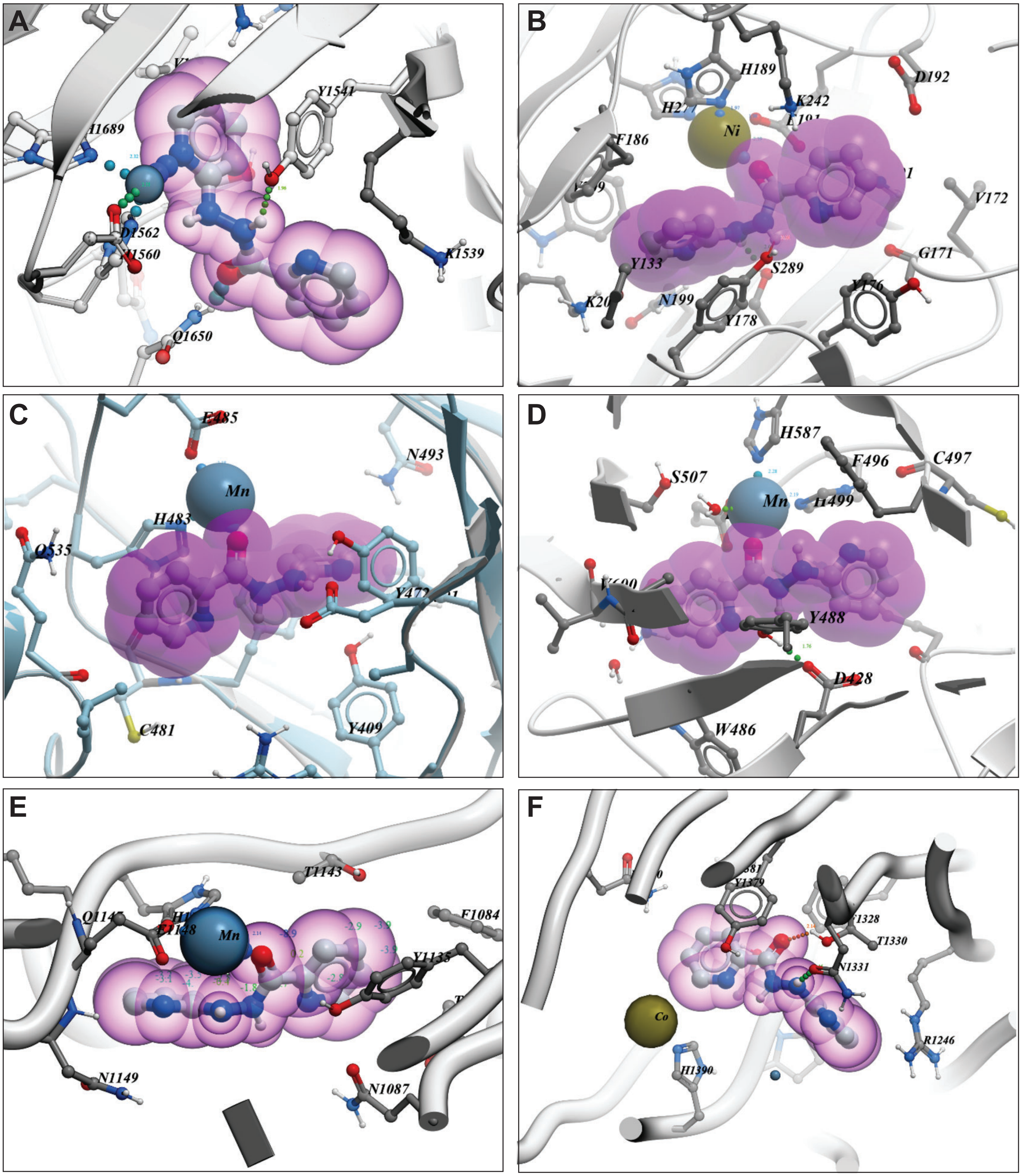

Supplementary Figure 7. P3FI-90 docked into the structures of KDM3B (**A**, PDB: 5RAN), KDM4B (**B**, PDB: 7JM5), KDM5A (**C**, PDB:6QD4), KDM5B (**D**, PDB:5A3T), KDM6A (**E**, PDB:6G8F), KDM6B (**F**, PDB:4ASK). The compound was docked into active sites of corresponding enzymes using ICM-Pro software under conditions of flexible receptor docking. Binding modes predict interaction with a metal ion in all cases except for KDM6B. However, the modes of interaction differ. KDM6B forms no bonds through Co^{2+} of the active site but rather two hydrogen bonds with the compound through side chains of Thr 1330 and Asn 1331. In case of KDM3B, interaction with Mn^{2+}occurs through the nitrogen of compound's pyridine moiety, like the ligands from experimental structures deposited to PDB, 5RAM, 5RAN, 5RAO, 5RAZ and 6RBJ. In all other enzymes the carbonyl group of the compound interacts with the metal ion. In addition to interaction with the Mn2+, KDM3B forms hydrogen bonds with P3FI-90 through the side chains of Gln 1650, Tyr 1541 and Asp 1562. KDM4B forms only one, through Ser289, and KDM5A, KDM5B and KDM6A do not appear to form any.

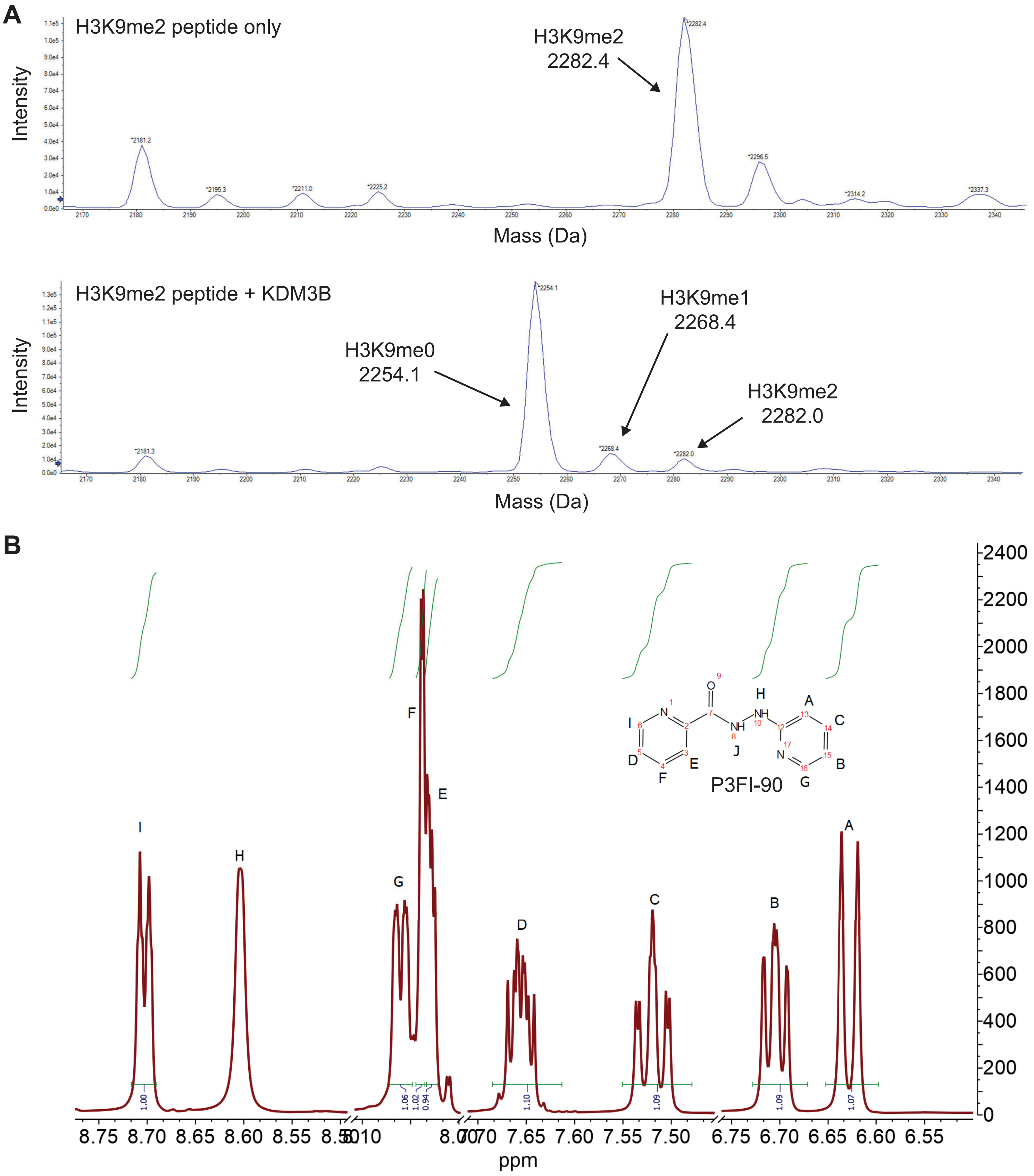

Supplementary Figure 8. A, Testing enzymatic function of full length KDM3B in demethylating H3K9me2. Reconstructed mass spectrum showing methyl group removal after incubation with KDM3B for 3 hr at room temperature. **B,** H^1 NMR of P3FI-90 to assign H peaks to specific locations of P3FI-90.

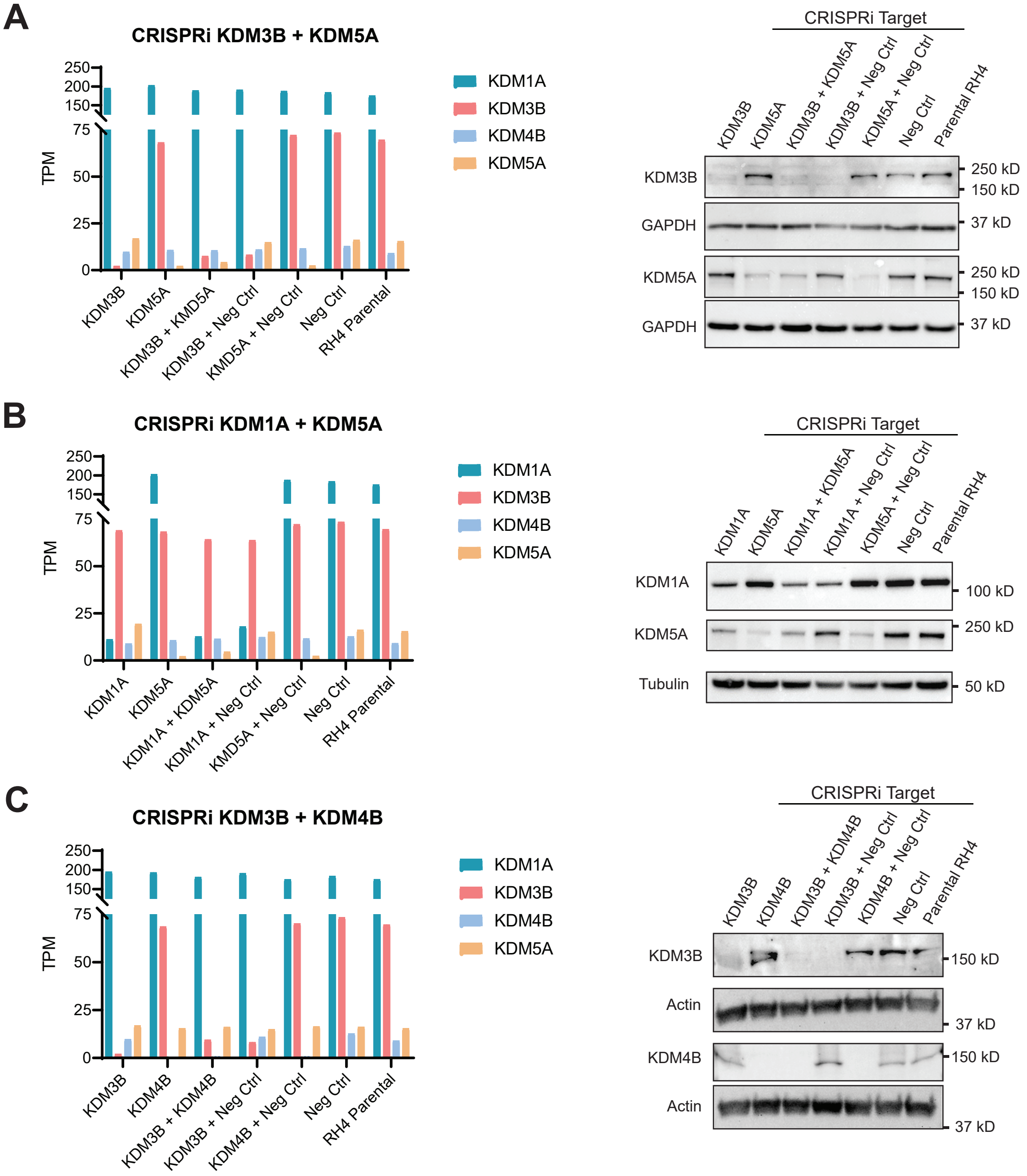

D

	shP3F	CRISPRi KDM3B	CRISPRi KDM1A	CRISPRi KDM4B	CRISPRi KDM5A	CRISPRi 1A + 3B	CRISPRi 3B + 5A	CRISPRi 1A + 5A
GRYDER_PAX3FOXO1_ENHANCERS_KO_DOWN	-3.28169	-2.18556	-2.09078			-2.19032		
EBAUER_TARGETS_OF_PAX3_FOXO1_FUSION_DN	-2.48828	-2.04518			-1.74752	-1.81857		
GRYDER_PAX3FOXO1_JQ1	-2.48786	-2.55892			-1.61203	-1.62517	-2.09822	
DAVICIONI_RHABDOMYOSARCOMA_PAX_FOXO1_FUSION_UP	-2.47957	-2.02431	-1.99579		-2.0722	-1.71627		
TARGETS_OF_PAX3_FOXO1_MELTZER_CHIPSEQ	-2.46639	-1.9763	-2.019			-2.11519		-1.48917
DAVICIONI_TARGETS_OF_PAX_FOXO1_FUSIONS_UP	-2.35355	-1.66153						
BEGUM_TARGETS_OF_PAX3_FOXO1_FUSION_UP	-2.27047							
DAVICIONI_PAX_FOXO1_SIGNATURE_IN_ARMS_UP	-2.25205	-1.9808	-1.999		-1.74967	-1.77715		
CORRELATED_0.5_WITH_TFAP2B_PAX_FOXO1_EXPRESSION_SIGNATURE_UP	-2.21506	-2.24584	-1.67116		-2.06454	-1.90764		
DAVICIONI_MOLECULAR_ARMS_VS_ERMS_UP	-1.76972	-1.68674	-1.51512			-1.71228		
GRYDER_PAX3FOXO1_CRC	-1.74453	-1.64091				-1.57162		
TRICHE_PAX3FOXO1A_TUMOR_UP	-1.74401					-1.70174		
GRYDER_PAX3FOXO1_TOP_ENHANCERS	-1.70565	-1.72405	-1.56949			-1.7125		
Total NES Score	-29.26	-21.73	-12.86	0.00	-9.25	-19.85	-2.10	-1.49

Supplementary Figure 9. CRISPRi knockdown of KDMs. **A,** Transcript in Transcripts per Million (TPM) (left) and western blot validation (right) of CRISPRi targeting KDM3B and KDM5A. One representative experiment from n =2 is shown. **B,** Transcript in TPM and western blot validation of CRISPRi targeting KDM1A and KDM5A. One representative experiment from n =2 is shown. **C,** Transcript in TPM and western blot validation of CRISPRi targeting KDM3B and KDM4B. One representative experiment from n =2 is shown. **D,** RNA-seq GSEA of PAX3-FOXO1 signatures for CRISPRi knockdown of KDMs compared to shP3F knockdown.

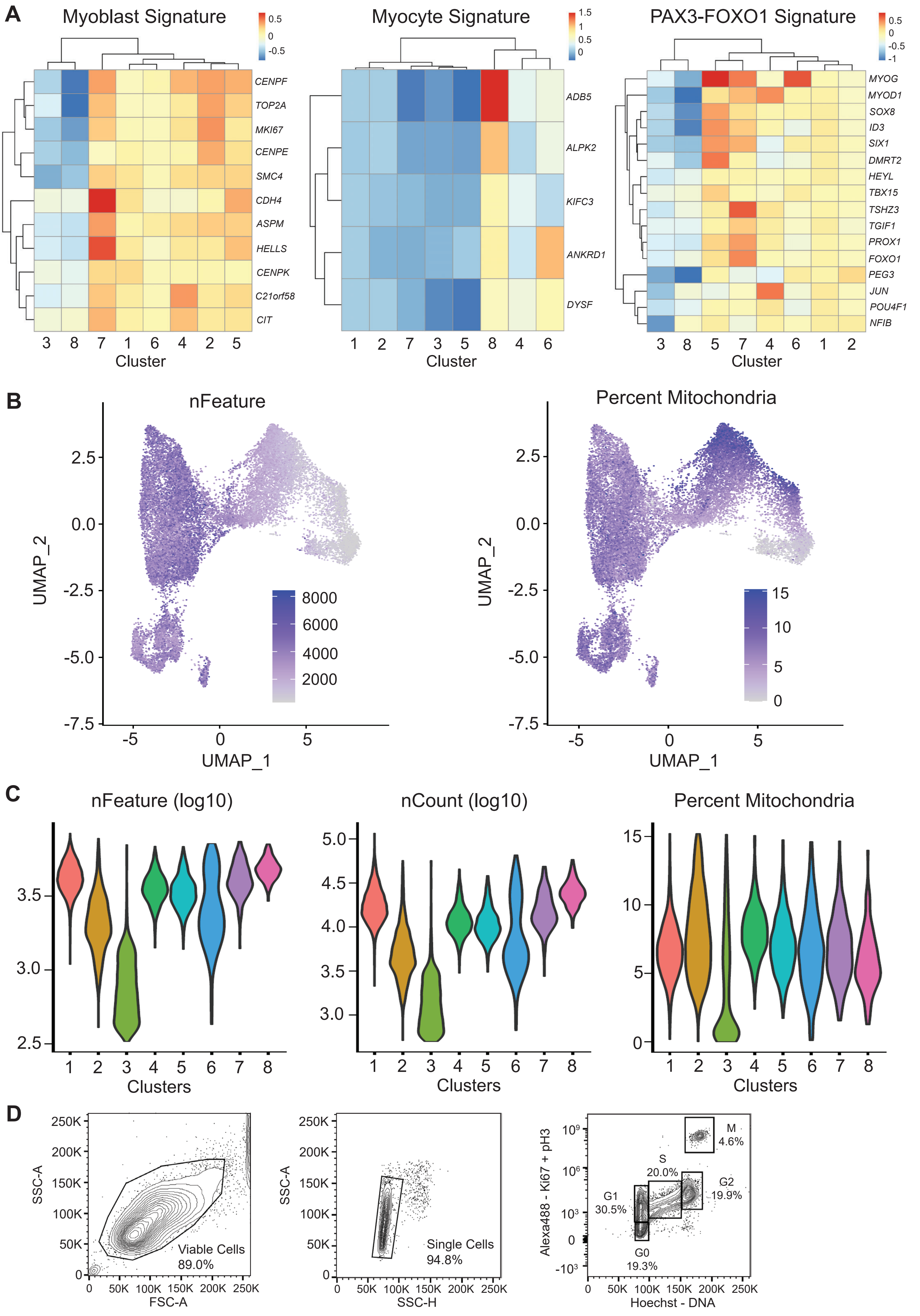
A
Myoblast Signature
0.5
0
-0.5
CENPF
TOP2A
MKI67
CENPE
SMC4
CDH4
ASPM
HELLS
CENPK
C21orf58
CIT
3 8 7 1 6 4 2 5
Cluster
Myocyte Signature
1.5
1
0.5
0
ADB5
ALPK2
KIFC3
ANKRD1
DYSF
1 2 7 3 5 8 4 6
Cluster
PAX3-FOXO1 Signature
0.5
0
-0.5
-1
MYOG
MYOD1
SOX8
ID3
SIX1
DMRT2
HEYL
TBX15
TSHZ3
TGIF1
PROX1
FOXO1
PEG3
JUN
POU4F1
NFIB
3 8 5 7 4 6 1 2
Cluster
B
nFeature
UMAP_2
UMAP_1
2.5
0.0
-2.5
-5.0
-7.5
-5
0
5
8000
6000
4000
2000
Percent Mitochondria
15
10
5
0
C
nFeature (log10)
3.5
3.0
2.5
1 2 3 4 5 6 7 8
Clusters
nCount (log10)
5.0
4.5
4.0
3.5
3.0
Percent Mitochondria
15
10
5
0
D
SSC-A
FSC-A
250K
200K
150K
100K
50K
0
50K
100K
150K
200K
250K
Viable Cells
89.0%
SSC-H
Single Cells
94.8%
Alexa488 - Ki67 + pH3
Hoechst - DNA
10^9
10^6
10^3
-10^3
M
4.6%
S
20.0%
G2
19.9%
G1
30.5%
G0
19.3%

Supplementary Figure 10. A, Heatmap of scRNA-seq clusters based on leading edge genes from myoblast, myocyte, PAX3-FOXO1 signatures. n=1. **B,** UMAPs of features per cell and percent mitochondria. **C,** Violin plot of features per cell, counts per cell, and percent mitochondria. **D,** Gating strategy for cell cycle analysis.

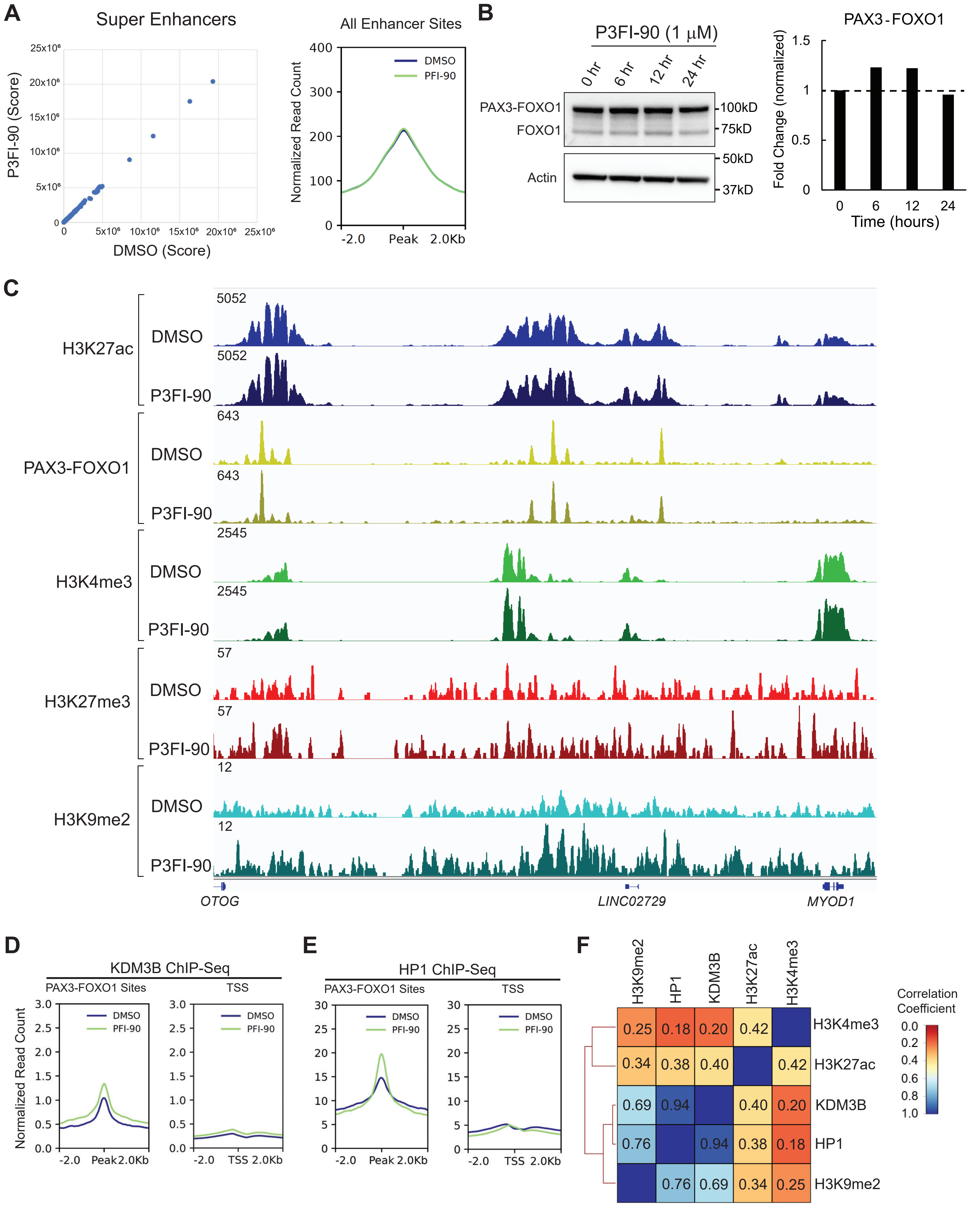
A
Super Enhancers
P3FI-90 (Score)
DMSO (Score)
All Enhancer Sites
Normalized Read Count
DMSO
PFI-90
-2.0
Peak
2.0Kb
B
P3FI-90 (1 μM)
0 hr
6 hr
12 hr
24 hr
PAX3-FOXO1
FOXO1
Actin
100kD
75kD
50kD
37kD
PAX3-FOXO1
Fold Change (normalized)
Time (hours)
C
H3K27ac
PAX3-FOXO1
H3K4me3
H3K27me3
H3K9me2
DMSO
P3FI-90
5052
643
2545
57
12
OTOG
LINC02729
MYOD1
D
KDM3B ChIP-Seq
PAX3-FOXO1 Sites
TSS
Normalized Read Count
E
HP1 ChIP-Seq
PAX3-FOXO1 Sites
TSS
F
H3K9me2
HP1
KDM3B
H3K27ac
H3K4me3
Correlation Coefficient
H3K4me3: 0.25 0.18 0.20 0.42
H3K27ac: 0.34 0.38 0.40 0.42
KDM3B: 0.69 0.94 0.40 0.20
HP1: 0.76 0.94 0.38 0.18
H3K9me2: 0.76 0.69 0.34 0.25

Supplementary Figure 11. A, Plot of all super enhancers using H3K27ac ChIP-seq results by ROSE analysis with each super enhancer plotted by score from P3FI-90 (1µM) vs DMSO at the 24 hr time point. Profile of H3K27ac at all enhancer sites. **B,** PAX3-FOXO1 western blot and quantitation normalized to actin loading control and time 0 in the RH4 cell line at various time points after treatment with P3FI-90 1µM. One representative experiment from n =2 is shown. **C,** IGV view of histone marks and PAX3-FOXO1 24 hr after treatment with P3FI-90 (1µM) vs DMSO. **D**, ChIP-seq profile of KDM3B at TSS and P3F sites. **E**, ChIP-seq profile of HP1 at TSS and P3F sites. **F**, Correlation heatmap of ChIP-seq results for KDM3B, HP1, and H3K9me2 showing that they colocalize. Source data are provided as a Source Data file.

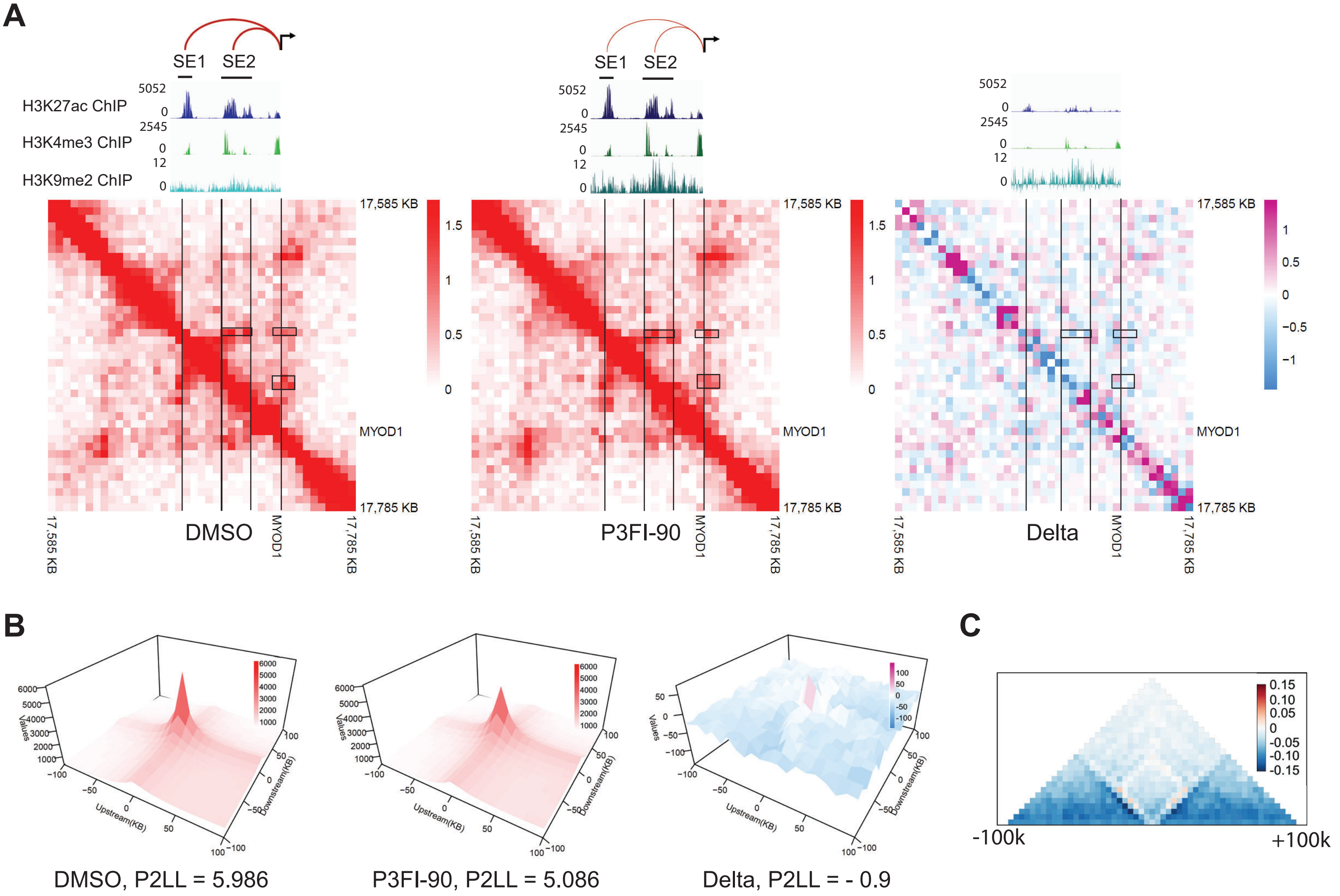

Supplementary Figure 12. HiC analysis of RH4 cells treated for 24 hr with P3FI-90 (1µM) vs DMSO. **A,** MYOD1 loop analysis with ChIP-seq track of H3K27ac, H3K4me3, and H3K9me2. Super Enhancer (SE) **B,** Genome wide aggregate peak analysis (APA) loop analysis. Peak to Lower Left (P2LL) **C,** Genome-wide delta TAD score of P3FI-90 vs DMSO.

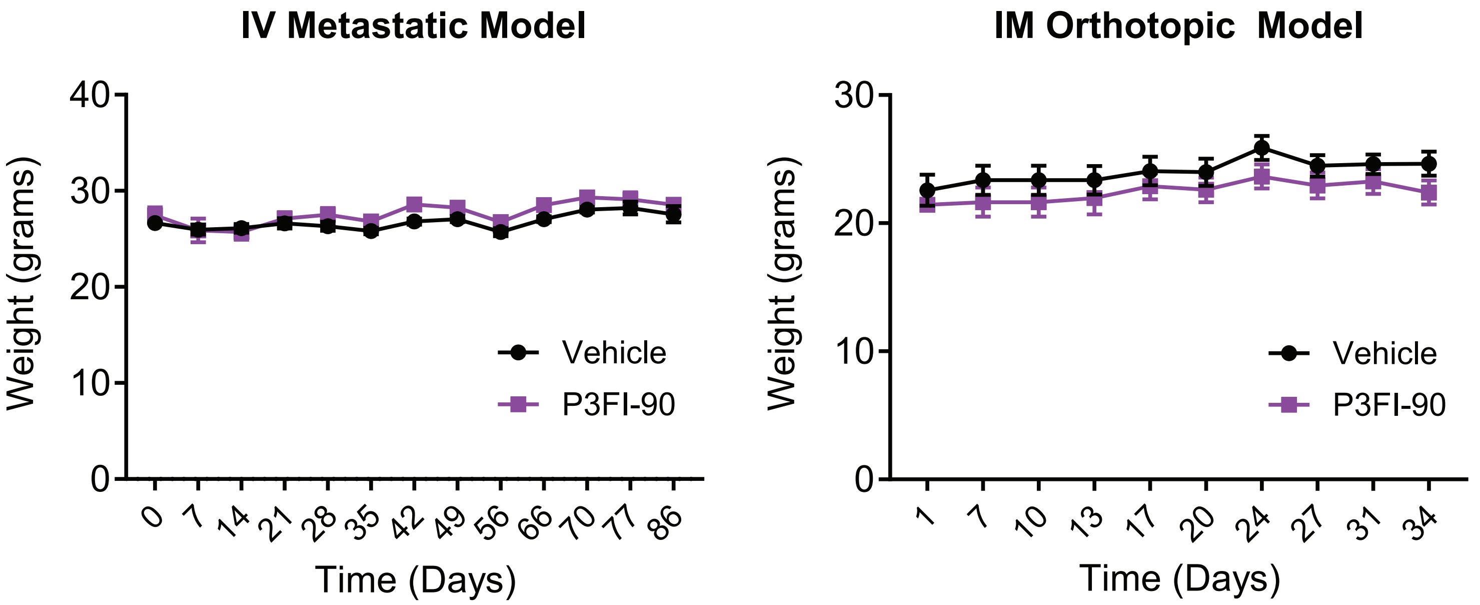

Supplementary Figure 13. Weight measurement during treatment of mice with DMSO vs P3FI-90 in IV metastatic model and IM orthotopic model. Data presented as mean values +/- SEM. n=5 for DMSO and n=4 for P3FI-90 in IV metastatic model and n=4 for both groups in IM orthotopic model. Source data are provided as a Source Data file.

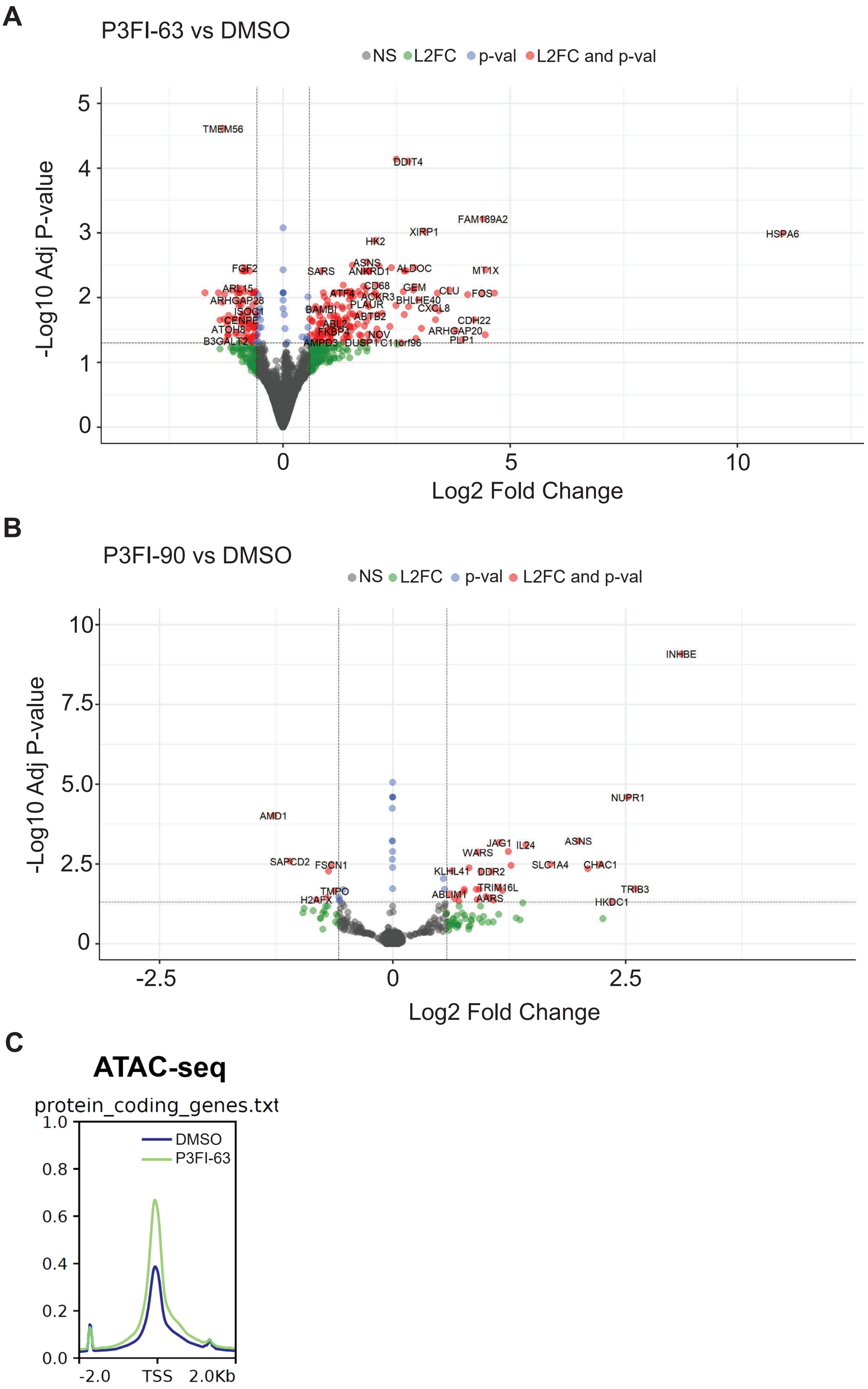
A
P3FI-63 vs DMSO
NS L2FC p-val L2FC and p-val
-Log10 Adj P-value
Log2 Fold Change
TMEM56
DDIT4
FAM189A2
XIRP1
HSPA6
HK2
FGF2
SARS
ASNS
ANKRD1
ALDOC
MT1X
ARL15
CD68
GEM
CLU
FOS
ARHGAP28
ATF4
ACKR3
BHLHE40
ISOC1
BAMBI
PLAUR
CXCL8
CENPE
ABTB2
CDH22
ATOH8
ARL2
FKBP4
ARHGAP20
NOV
PLP1
B3GALT2
AMPD3
DUSP1
C11orf96
B
P3FI-90 vs DMSO
NS L2FC p-val L2FC and p-val
-Log10 Adj P-value
Log2 Fold Change
INHBE
NUPR1
AMD1
JAG1
IL24
ASNS
WARS
SAPCD2
FSCN1
SLC1A4
CHAC1
KLHL41
DDR2
TRIM16L
TRIB3
TMPO
ABLIM1
AARS
H2AFX
HKDC1
C
ATAC-seq
protein_coding_genes.txt
DMSO
P3FI-63
-2.0
TSS
2.0Kb

Supplementary Figure 14. A, QC volcano plot of RNA-seq triplicate sample of P3FI-63 vs DMSO. **B**, QC volcano plot of RNA-seq triplicate sample of P3FI-90 vs DMSO. **C,** QC profile of ATAC-seq after treatment with P3FI-63 vs DMSO at the transcription start site (TSS) showing enrichment.
