# Supplementary material for: KDM3B inhibitors disrupt the oncogenic activity of PAX3-FOXO1 in fusion-positive rhabdomyosarcoma
Source: Nat Commun. 2024 Feb 24;15:1703. doi: 10.1038/s41467-024-45902-y (PMC10894237; doi:10.1038/s41467-024-45902-y)
Supplement: Supplementary file 3 — Description of Additional Supplementary Files [file 41467_2024_45902_MOESM3_ESM.pdf]

File Name: Supplementary Data 1
Description:
Tab 1: 64 compounds from secondary screen.
Tab 2: Similarity Ensemble Approach analysis of P3FI-63.
Tab 3: ATAC-seq GSEA for P3FI-63 vs DMSO.
Tab 4: RNA-seq GSEA for JIB-04 vs DMSO.
Tab 5: RNA-seq GSEA for GSK-J4 vs DMSO.
Tab 6: RNA-seq GSEA for GSK-690 vs DMSO.
Tab 7: ALK-luciferase and CMV-luciferase assay testing P3FI-63 analogs.
Tab 8: In Silico Docking analysis of P3FI-90 with KDMs.

File Name: Supplementary Data 2
Description:
Tab 1: RNA-seq GSEA of downregulated PAX3-FOXO1 gene sets of 7 compounds.
Tab 2: RNA-seq GSEA for shPAX3-FOXO1 vs shScramble.
Tab 3: RNA-seq GSEA for P3FI-63 (UPCMLD0ENAT5834780) vs DMSO.
Tab 4: RNA-seq GSEA for KUC102648N vs DMSO.
Tab 5: RNA-seq GSEA for NP005584 vs DMSO.
Tab 6: RNA-seq GSEA for KUC100517N vs DMSO.
Tab 7: RNA-seq GSEA for 78902794023 vs DMSO.
Tab 8: RNA-seq GSEA for 78800610218 vs DMSO.
Tab 9: RNA-seq GSEA for IMMLG5770 vs DMSO.

File Name: Supplementary Data 3
Description:
Tab 1: RNA-seq GSEA for P3FI-63 vs DMSO.
Tab 2: RNA-seq GSEA for P3FI-90 vs DMSO.
Tab 3: RNA-seq GSEA for CRISPRi knockdown of KDM3B vs Control.
Tab 4: RNA-seq GSEA for CRISPRi knockdown of KDM1A vs Control.
Tab 5: RNA-seq GSEA for CRISPRi knockdown of KDM4B vs Control.
Tab 6: RNA-seq GSEA for CRISPRi knockdown of KDM5A vs Control.
Tab 7: RNA-seq GSEA for CRISPRi knockdown of KDM1A + 3B vs Control.
Tab 8: RNA-seq GSEA for CRISPRi knockdown of KDM3B + 4B vs Control.
Tab 9: RNA-seq GSEA for CRISPRi knockdown of KDM3B + 5A vs Control.
Tab 10: RNA-seq GSEA for CRISPRi knockdown of KDM1A + 5A vs Control.

File Name: Supplementary Data 4
Description:
Tab 1: Myoblost gene set from Patel et al (Developmental Cell 2022) scRNA-seq manuscript.
Tab 2: Myocyte gene set from Patel et al (Developmental Cell 2022) scRNA-seq manuscript.
Tab 3: scRNA-seq cluster p-values for Figure 4c.
Tab 4: scRNA-seq cluster 1 vs rest gene list.
Tab 5: scRNA-seq cluster 2 vs rest gene list.
Tab 6: scRNA-seq cluster 3 vs rest gene list.
Tab 7: scRNA-seq cluster 4 vs rest gene list.
Tab 8: scRNA-seq cluster 5 vs rest gene list.
Tab 9: scRNA-seq cluster 6 vs rest gene list.
Tab 10: scRNA-seq cluster 7 vs rest gene list.
Tab 11: scRNA-seq cluster 8 vs rest gene list.

File Name: Supplementary Data 5
Description:
Tab 1: ChIP-seq differential peak analysis for H3K9me2 vs DMSO.
Tab 2: CHEA analysis of H3K9me2 differential peaks.
Tab 3: ChIP-seq differential peak analysis for H3K4me3 vs DMSO.
Tab 4: Gene ontology analysis of H3K4me3 differential peaks.
Tab 5: GSEA analysis of downregulated TADs and Loops P3FI-90 vs DMSO.
Tab 6: GSEA analysis of RNA Pol2 ChIP for P3FI-90 vs DMSO.
Tab 7: GSEA analysis of RNA Pol2 Ser5 ChIP for P3FI-90 vs DMSO.

File Name: Supplementary Data 6
Description:
Tab 1: Mouse Tumor RNA-seq GSEA for P3FI-90 vs DMSO group analysis n=2.
Tab 2: Mouse #920 Tumor RNA-seq GSEA for P3FI-90 vs DMSO.
Tab 3: Mouse #924 Tumor RNA-seq GSEA for P3FI-90 vs DMSO.

File Name: Supplementary Data 7
Description:
Tab 1: Table of antibodies used in the study.
Tab 2: QC of next generation sequencing experiments in the study.
Tab 3: GSEA gene sets and references.
