# Supplementary material for: KDM3B inhibitors disrupt the oncogenic activity of PAX3-FOXO1 in fusion-positive rhabdomyosarcoma
Source: Nat Commun. 2024 Feb 24;15:1703. doi: 10.1038/s41467-024-45902-y (PMC10894237; doi:10.1038/s41467-024-45902-y)
Supplement: Supplementary file 11 — Reporting Summary [file 41467_2024_45902_MOESM11_ESM.pdf]

nature portfolio

Corresponding author(s): Javed Khan, MD
Yong Yean Kim, MD/PhD

Last updated by author(s): Jan 30, 2024

Reporting Summary

Nature Portfolio wishes to improve the reproducibility of the work that we publish. This form provides structure for consistency and transparency in reporting. For further information on Nature Portfolio policies, see our Editorial Policies and the Editorial Policy Checklist.

Statistics

For all statistical analyses, confirm that the following items are present in the figure legend, table legend, main text, or Methods section.

n/a	Confirmed	
☐	☒	The exact sample size (*n*) for each experimental group/condition, given as a discrete number and unit of measurement
☐	☒	A statement on whether measurements were taken from distinct samples or whether the same sample was measured repeatedly
☐	☒	The statistical test(s) used AND whether they are one- or two-sided<br>*Only common tests should be described solely by name; describe more complex techniques in the Methods section.*
☒	☐	A description of all covariates tested
☐	☒	A description of any assumptions or corrections, such as tests of normality and adjustment for multiple comparisons
☐	☒	A full description of the statistical parameters including central tendency (e.g. means) or other basic estimates (e.g. regression coefficient) AND variation (e.g. standard deviation) or associated estimates of uncertainty (e.g. confidence intervals)
☐	☒	For null hypothesis testing, the test statistic (e.g. *F*, *t*, *r*) with confidence intervals, effect sizes, degrees of freedom and *P* value noted<br>*Give P values as exact values whenever suitable.*
☒	☐	For Bayesian analysis, information on the choice of priors and Markov chain Monte Carlo settings
☒	☐	For hierarchical and complex designs, identification of the appropriate level for tests and full reporting of outcomes
☒	☐	Estimates of effect sizes (e.g. Cohen's *d*, Pearson's *r*), indicating how they were calculated

Our web collection on statistics for biologists contains articles on many of the points above.

Software and code

Policy information about availability of computer code

Data collection

Illumina NovaSeq 2000 system was used for scRNA-seq and Hi-C.
Illumina NextSeq 500 system was used for ChIP-Seq, RNA-seq, ATAC-seq, and CRIPSR experiments.
BioRad ChemiDoc MP 2.3.0.07 was used to obtain images of Western Blots.
Incucyte Zoom life cell imaging system was used for IC50 experiments.
BD LSRFortessa™ Cell Analyzer was used for flow analysis
Tecan Spark was used to analyze absorbance and fluorescence.
SCIEX X500B Q-TOF mass spectrometer coupled with Exion UHPLC was used to obtain mass spec data.
NMR spectra was acquired on Bruker AVANCE III 500 MHz spectrometer equipped with a Bruker TCI cryogenically cooled probe.
SPR was performed on Biacore T200 using CM5 chip.
Molecular docking experiment was using ICM-Pro software from Molsoft
IVIS Lumina III In Vivo Imaging system

Data analysis

RNA-seq: Reads were mapped to the human genome build hg19 by STAR (https://github.com/alexdobin/STAR) and quantified using RSEM (https://deweylab.github.io/RSEM/). DESeq2 (1.26.0) was used in differential gene analysis. Gene set enrichment was assessed using GSEA software (https://www.gsea-msigdb.org/gsea/). Leading edge analysis was performed using the same GSEA.
ChIP-seq and ATAC-seq: Data was mapped to hg19 using BWA. For samples with spike in, we additionally mapped spike in reads to dm3 using BWA (version 0.7.17), and normalized human reads to million-mapped Drosophila reads (RRPM, reference normalize reads per million). Peaks were called using MACS2.0, with stringency thresholds of p = 0.0000001 and filtered to remove ENCODE blacklisted regions (satellite repeats and spuriously over-mapped regions). Software tools used include deepTools, bedtools, GSEA. A custom code for ChIP-seq and ATAC-seq analyses is available on github at https://github.com/CBIIT/ChIP_seq. Genome tracks were visualized in IGV (version 2.12.3). ChIP-seq which

were performed in triplicates were analyzed using DiffBind's dba.count.
scRNA-seq: Demultiplexing, barcoded processing, gene counting and aggregation were made using the Cell Ranger software v6.1.2. Seurat version 4.0 was used to analyze the data.
Hi-C: Data was mapped to hg19 using omni-c_qc.sh from Dovetail Github (https://github.com/dovetail-genomics). Hi-C Technical repliaces were merged using Pairtools' merge. Software tools used include FAN-C, GeneOverlap, p.adjust, bedtools, hicFindTADs, hicDifferentialTAD, and Juicer's apa.
CRISPR: MAGeCK was used to analyze the data.
GraphPad Prism 8.0, R version 3.6.1, or GSEA 4.2.3 were used for statistical analysis.
BioRad Image Lab 6.1 software was used to quantitate Western blot bands.
FlowJo software was used for data analysis.
Mass Spec data was analyzed using Explorer and BioToolKit from SCIEX.
NMR data was analyzed and images generated using MestReNova software version 14.2.0.
Molecular docking experiment analysis was performed using ICM-Pro from Molsoft using PoketFinder and Radial Convolutioinal Neural Net.
IVIS live mouse imaging data was analyzed using Living Image version 4.5

For manuscripts utilizing custom algorithms or software that are central to the research but not yet described in published literature, software must be made available to editors and reviewers. We strongly encourage code deposition in a community repository (e.g. GitHub). See the Nature Portfolio guidelines for submitting code & software for further information.

Data

Policy information about availability of data

All manuscripts must include a data availability statement. This statement should provide the following information, where applicable:

- Accession codes, unique identifiers, or web links for publicly available datasets
- A description of any restrictions on data availability
- For clinical datasets or third party data, please ensure that the statement adheres to our policy

All sequencing files are available on GEO under the accession number GSE219199 [https://www.ncbi.nlm.nih.gov/geo/query/acc.cgi?acc=GSE219199]. Sequencing files both raw fastq files and processed files included are RNA-seq (TPM matrix text file), ChIP-seq (Peak call bed file), ATAC-seq (peak call bed file), scRNA-seq (barcodes, features, and matrix files), and HiC (merged hic files). Protein crystal structures were accessed through the PDB database. https://www.rcsb.org/. Enricher database was utilized for analysis of ChIP results: https://maayanlab.cloud/Enrichr/.26 GO database was utilized for analysis of ChIP results: https://geneontology.org/. Majority of the gene sets were obtained from GSEA website: https://www.gsea-msigdb.org/gsea/index.jsp. Gene sets from papers are referenced in the Supplementary Data 7 Tab3. Source data are provided with this paper. Publicly available scRNA-seq data defining differential genes for myogenesis were obtained from Patel et al publication Supplemental Table S3.23 Publicly available ChIP-seq data used in this study are available in the GEO database under accession codes GSE83728 [https://www.ncbi.nlm.nih.gov/geo/query/acc.cgi?acc=GSE83728] and GSE116344 [https://www.ncbi.nlm.nih.gov/geo/query/acc.cgi?acc=GSE116344].6, 24 The remaining data are available within the Article, Supplementary Information or Source Data file.

Research involving human participants, their data, or biological material

Policy information about studies with human participants or human data. See also policy information about sex, gender (identity/presentation), and sexual orientation and race, ethnicity and racism.

Reporting on sex and gender	No human participants were involved in this study.
Reporting on race, ethnicity, or other socially relevant groupings	No human participants were involved in this study.
Population characteristics	No human participants were involved in this study.
Recruitment	No human participants were involved in this study.
Ethics oversight	No human participants were involved in this study.

Note that full information on the approval of the study protocol must also be provided in the manuscript.

Field-specific reporting

Please select the one below that is the best fit for your research. If you are not sure, read the appropriate sections before making your selection.

[x] Life sciences   [ ] Behavioural & social sciences   [ ] Ecological, evolutionary & environmental sciences

For a reference copy of the document with all sections, see nature.com/documents/nr-reporting-summary-flat.pdf

Life sciences study design

All studies must disclose on these points even when the disclosure is negative.

Sample size	Since P3FI-90 is a novel compound, there is no prior information available for power analysis with this compound. Our prior experience using fusion positive rhabdomyosarcoma xenograft mouse models in small molecule testing indicated that sample size of 5 is sufficient to detect reduction of tumor size > 40% at p < 0.05 with probability of 80%. (Gryder, B. E., et al (2017). PAX3-FOXO1 Establishes Myogenic Super

Enhancers and Confers BET Bromodomain Vulnerability. Cancer discovery, 7(8), 884–899.) For in vitro experiments, no sample size calculation was performed.

Data exclusions

One mice was excluded due to death after randomization during anesthesia.

Replication

The mouse xenograft experiment was performed once as orthotopic intra-muscular model and once as metastatic intra-vascular model both with delay in tumor progression. This replication provided reproducibility as well as provided efficacy in both primary localized disease and metastatic disease.

Randomization

All tumor bearing mice were placed into one cage and allowed to mix. Then mice were randomly selected one at a time and placed into cage for control versus treatment in alternating order. In vitro studies were performed using cell lines and we did not randomize. Covariates were controlled by performing the experiment at the same time using the same growth media, flasks, reagents, kits, data collecting machine.

Blinding

Investigators were not blinded to treatment versus control. You raise a valid concern. However, this has not been a standard practice within the NIH IACUC.

Reporting for specific materials, systems and methods

We require information from authors about some types of materials, experimental systems and methods used in many studies. Here, indicate whether each material, system or method listed is relevant to your study. If you are not sure if a list item applies to your research, read the appropriate section before selecting a response.

Materials & experimental systems

n/a	Involved in the study
☐	☒ Antibodies
☐	☒ Eukaryotic cell lines
☒	☐ Palaeontology and archaeology
☐	☒ Animals and other organisms
☒	☐ Clinical data
☒	☐ Dual use research of concern
☒	☐ Plants

Methods

n/a	Involved in the study
☐	☒ ChIP-seq
☐	☒ Flow cytometry
☒	☐ MRI-based neuroimaging

Antibodies

Antibodies used

Antibody Target, Source, Cat#, Application, Dilution/Conc.
PAX3-FOXO1, In House, None, None, ChIP, 0.1 ug/uL. (Azorsa DO, et al. Mod Pathol, 2021)
FOXO1 (C29H4), Cell Signaling, 2880, Western, 1:2000.
H3K9me2, Abcam, ab1220, Western, ChIP, 1:5000, 0.03 µg/µL
H3K4me3, Active motif, 39159, Western, ChIP, 1:4000, 0.03 µg/µL
H3K27me3, Active motif, 39155, Western, ChIP, 1:4000, 0.03 µg/µL
H3K27ac, Active motif, 39133, ChIP, 1:30
Total RNA Pol2, Millipore, 05-623, ChIP, 1:26
RNA Pol2 Ser5, Cell Signaling, 13523, ChIP, 1:43
RNA Pol2 Ser2, Cell Signaling, 13499, ChIP, 1:43
HP1, Cell Signaling, 2616, ChIP, 1:20
KDM3B, Cell Signaling, 3100, ChIP, 1:33.3
H3 Total Histone, Cell Signaling, 9715, Western, 1:5000
MYOG, Invitrogen, MA1-41042, Western, 1:500
Total PARP, Cell Signaling, 9542, Western, 1:1000
KDM1A, Cell Signaling,, 2184S, Western, 1:1000
KDM3B, Cell Signaling, 5377T, Western, 1:1000
KDM4B, Cell Signaling, 8639S, Western, 1:1000
KDM5A, Cell Signaling, 3876T, Western, 1:1000
Tubulin, Abcam, ab7291, Western, 1:5000
Actin-HRP, Santa Cruz, sc-47778, Western, 1:10000
GAPDH-HRP, Santa Cruz, sc-47724, Western, 1:10000
anti-mouse-HRP, Santa Cruz, sc-2005, Western, 1:5000
anti-rabbit-HRP, Santa Cruz, sc-2004, Western, 1:5000
Ki67, Invitrogen, MA5-14520, Flow Cytometry, 1:150
pH3, Biolegend, 650802, Flow Cytometry, 1:40
anti-rabbit-Alexa488, Invitrogen, A-11034, Flow Cytometry, 1:200

Validation

For the in-house PAX3-FOXO1 antibody, please refer the reference (Azorsa DO, et al. Mod Pathol, 2021, PMID: 33299109) for validation.
Cell Signaling antibody Validation is available on their website https://www.cellsignal.com/.
FOXO1 #2880: FoxO1 (C29H4) Rabbit mAb detects endogenous levels of total FoxO1 protein. The antibody does not detect

exogenously expressed family members FoxO3a or FoxO4. Species Reactivity: Human, Mouse, Rat, Monkey.
RNA Pol2 Ser5 #13523: Phospho-Rpb1 CTD (Ser5) (D9N5I) Rabbit mAb recognizes endogenous levels of Rpb1 only when the carboxy-terminal domain (CTD) heptapeptide repeat [Tyr1, Ser2, Pro3, Thr4, Ser5, Pro6, Ser7] is phosphorylated at Ser5. This antibody does not cross-react with Rpb1 CTD phosphorylated at Ser2 or Ser7. Species Reactivity: Human, Mouse, Rat, Monkey.
RNA Pol2 Ser2 #13499: Phospho-Rpb1 CTD (Ser2) (E1Z3G) Rabbit mAb recognizes endogenous levels of Rpb1 only when the carboxy-terminal domain (CTD) heptapeptide repeat [Tyr1, Ser2, Pro3, Thr4, Ser5, Pro6, Ser7] is phosphorylated at Ser2. This antibody does not cross-react with Rpb1 CTD phosphorylated at Ser5 or Ser7. Species Reactivity: Human, Mouse, Rat, Monkey.
H3 Total Histone #9715:Histone H3 Antibody detects endogenous levels of total histone H3 protein. This antibody does not cross-react with other histones. Species Reactivity: Human, Mouse, Rat, Monkey, Zebrafish, Bovine, Pig.
HP1 #2616: HP1 alpha antibody detects endogenous levels of total HP1alpha protein. The antibody does not cross-react with HP1 beta or HP1 gamma proteins.
KDM3B #3100: JMJD1B (C6D12) Rabbit mAb detects endogenous levels of JMJD1B protein (all three isoforms). This antibody does not cross react with other Jumonji C proteins, including HR, JMJD1A and JMJD1C.
Total PARP #9542:PARP Antibody detects endogenous levels of full length PARP1 (116 kDa), as well as the large fragment (89 kDa) of PARP1 resulting from caspase cleavage. The antibody does not cross-react with related proteins or other PARP isoforms. Species Reactivity: Human, Mouse, Rat, Monkey.
KDM1A #2184: LSD1 (C69G12) Rabbit mAb detects endogenous levels of total LSD1 protein. Species Reactivity: Human, Mouse, Rat, Monkey.
KDM3B #5377: JMJD1B (6A1-1F5) Mouse mAb detects endogenous levels of total JMJD1B protein. Species Reactivity: Human, Mouse, Rat, Monkey.
KDM4B #8639:J MJD2B (D7E6) Rabbit mAb recognizes endogenous levels of total JMJD2B protein. This antibody does not cross-react with other Jumonji C proteins, including JMJD2A, JMJD2C, and JMJD2D. Species Reactivity: Human, Monkey.
KDM5A #3876: JARID1A (D28B10) Rabbit mAb detects endogenous levels of total JARID1A protein (both isoforms). The antibody does not cross-react with other JARID proteins, including JARID1B, JARID1C and JARID1D. Species Reactivity: Human, Mouse.
Millipore antibody Validation is available on their website https://www.emdmillipore.com/US/en/life-science-research/antibodies-assays/antibodies-overview/l6Wb.qB.p6cAAAFOKNAqQvST,nav.
Total RNA Pol2:Anti-RNA polymerase II Antibody, clone CTD4H8 is a high quality Mouse Monoclonal Antibody for the detection of RNA polymerase II and has been published in more than 40 citations and validated for use in ChIP & WB.
Abcam antibody validation is available on their website https://www.abcam.com/
H3K9me2 #ab1220: ChIP Grade. Suitable for: ICC/IF, WB, ELISA, IHC-P, ChIP. Reacts with: Cow, Human, Arabidopsis thaliana, Drosophila melanogaster, Rice, Recombinant fragment.
Tubulin #ab7291: Suitable for: Flow Cyt, ICC/IF, IHC-P, WB. Reacts with: Mouse, Rat, Human.
Active Motif antibody validation is available on their website https://www.activemotif.com/
H3K4me3 #39159: Applications Validated by Active Motif: ChIP: 3 - 5 µl per ChIP, ChIP-Seq: 3 µl each, ICC/IF: 1:500 - 1:1,000 dilution, WB: 1:500 - 1:2,000 dilution, **CUT&Tag: 1 µl per 50 µl reaction*.
H3K27me3 #39155: Applications Validated by Active Motif: ChIP: 5 - 10 µg per, ChIP ChIP-Seq: 5 µg each, ICC/IF: 2 µg/ml dilution, IHC (FFPE): 2 µg/ml dilution, WB*: 0.5 - 2 µg/ml dilution, CUT&Tag: 1 µg per 50 µl reaction*.
H3K27ac #39133: Applications Validated by Active Motif: ChIP: 10 µg per ChIP, ChIP-Seq: 5 µg each, ICC/IF: 1 - 5 µg/ml dilution, WB*: 0.1 - 1 µg/ml dilution, CUT&Tag: 1 µg per 50 µl reaction*
Invitrogen antibody validation is available on ThermoFisher website https://www.thermofisher.com/us/en/home/life-science/antibodies.html.
MYOG #MA1-41042: Application: Western Blot, IHC, ICC/IF. Species: Human, Mouse, Rat.
Ki-67 MA5-14520: Application: Western Blot, IHC, ICC/IF, Flow, Neutralization. Species: Human, Mouse, Rat, Non-human primate.
anti-rabbit-Alexa488 A-11034: Application: ICC/IF, Flow. Species: Rabbit.
Biolegend antibody validation is available on their website https://www.biolegend.com/
pH3 650802: The histone H3 pS10 antibody recognizes phosphorylation of human H3 protein at Ser10 residue and has been shown to be useful for Western blotting. Application: Western blot and IF.
Santa Cruz antibody validation is available on their website https://www.scbt.com/home/
GAPDH-HRP sc-47724:GAPDH Antibody (0411) is recommended for detection of GAPDH of human origin by WB, IP, IF and IHC(P); not recommended for detection of GAPDH of mouse or rat origin
anti-mouse-HRP sc-2005: recommended for use in Western blotting at a dilution of 1:500 - 1:10000. Starting dilution: 1:2000
anti-rabbit-HRP sc-2004: recommended for use in Western blotting at a dilution of 1:500 - 1:10000. Starting dilution: 1:5000
Actin-HRP sc-47778: beta Actin Antibody (C4) is recommended for detection of β-Actin of mouse, rat, human, avian, bovine, canine, porcine, rabbit, Dictyostelium discoideum and Physarum polycephalum origin by WB, IP, IF, IHC(P) and ELISA; may cross-react with all six known isoforms of Actin in higher vertebrates (including cytoplasmic β- and γ- Actin isoforms, skeletal, cardiac, and vascular α-Actin isoforms, and enteric γ-Actin isoform)

Eukaryotic cell lines

Policy information about cell lines and Sex and Gender in Research

Cell line source(s)	The cell line RH4 and RH30 were all provided by Dr. Peter Houghton, Greehey Children's Cancer Research Institute, San Antonio, Texas, USA. RD and CTR were provided by Dr. Lee Helman, Children's Hospital Los Angeles, CA, USA. SCMC was provided by Dr. Janet Shipley, Institute of Cancer Research, London, England. Ewing's sarcoma (TC-32 and A673) and osteosarcoma (OSA and HU09) cell lines were provided by Dr. Paul Meltzer, NCI, NIH, Bethesda, MD. Human fibroblast 7250 (CRL7250) cell lines was obtained through ATCC.
Authentication	Cell lines were authenticated using short tandem repeats analysis.
Mycoplasma contamination	All cell lines tested negative for Mycoplasma contamination.

Commonly misidentified lines (See ICLAC register)	No commonly misidentified cell lines were used in this study.

Animals and other research organisms

Policy information about studies involving animals; ARRIVE guidelines recommended for reporting animal research, and Sex and Gender in Research

Laboratory animals	Mice used in this study are NSG mice strain ID#005557 (Jackson Labs). Only female mice were used in the study. For the IV metastatic model, age of mice were 12 weeks old. For the IM orthotopic model, 7 week old mice were used.
Wild animals	This study did not involve wild animals
Reporting on sex	Female mice were used in this study which allows for housing of 5 mice per cage. Sex consideration was based on practical feasibility issues. This included aggressiveness of male mice with propensity to injure one another which is especially concerning in severely immunocompromised NSG mice. Mice are housed in IVM Microisolator conditions designated for immune compromised mice. Light cycle is 6am-6pm
Field-collected samples	This study did not involve field collected samples
Ethics oversight	This mouse study was reviewed and was approved by the NIH IACUC, protocol PB-057.

Note that full information on the approval of the study protocol must also be provided in the manuscript.

Plants

Seed stocks	N/A
Novel plant genotypes	N/A
Authentication	N/A

ChIP-seq

Data deposition

- [x] Confirm that both raw and final processed data have been deposited in a public database such as GEO.
- [x] Confirm that you have deposited or provided access to graph files (e.g. BED files) for the called peaks.

Data access links<br>*May remain private before publication.*	https://www.ncbi.nlm.nih.gov/geo/query/acc.cgi?acc=GSE219199
Files in database submission	Raw files will have "_fastq.gz" extension and processed files will have "_peaks.narrowPeak.nobl.bed.gz" for narrow peaks and "_peaks.braodPeak.nobl.bed.gz" for broad peaks.<br>Sample_RH4_DMSO_24h_PAX3FOXO1_048_C_HNJTGBGXB<br>Sample_RH4_PFI90_1_24h_PAX3FOXO1_048_C_HNJTGBGXB<br>Sample_RH4_DMSO_24h_H3K27ac_200125_C_H77JJBGXC<br>Sample_RH4_PFI90_1uM_24h_H3K27ac_200125_C_H77JJBGXC<br>Sample_RH4_DMSO_24h_H3K4me3_048_C_HNJTGBGXB<br>Sample_RH4_PFI90_1_24h_H3K4me3_048_C_HNJTGBGXB<br>Sample_RH4_DMSO_24h_H3K27me3_048_C_HNJTGBGXB<br>Sample_RH4_PFI90_1_24h_H3K27me3_048_C_HNJTGBGXB<br>Sample_RH4_DMSO_24h_H3K9me2_047_C_HNKLTBGXB<br>Sample_RH4_PFI90_1_24h_H3K9me2_047_C_HNKLTBGXB<br>Sample_RH4_DMSO_Pol2_201212_C_HV33VBGXG<br>Sample_RH4_PFI90_Pol2_201212_C_HV33VBGXG<br>Sample_RH4_DMSO_Pol2_ser5_201214_C_HV33VBGXG<br>Sample_RH4_PFI90_Pol2_ser5_201214_C_HV33VBGXG<br>Sample_RH4_DMSO_Pol2_ser2_201214_C_HV33VBGXG<br>Sample_RH4_PFI90_Pol2_ser2_201214_C_HV33VBGXG<br>Sample_RH4_DMSO_24h_H3K4me3_Rep3_C_HLTYHBGXM<br>Sample_RH4_DMSO_24h_H3K4me3_Rep4_C_H3NJCBGXK<br>Sample_RH4_PFI90_1uM_24h_H3K4me3_Rep3_C_HLTYHBGXM

Sample_RH4_PFI90_1uM_24h_H3K4me3_Rep4_C_H3NJCBGXK
Sample_RH4_DMSO_24h_H3K27ac_Rep2_C_AAAWF2NM5
Sample_RH4_DMSO_24h_H3K27ac_Rep4_C_H3NJCBGXK
Sample_RH4_PFI90_1uM_24h_H3K27ac_Rep2_C_AAAWF2NM5
Sample_RH4_PFI90_1uM_24h_H3K27ac_Rep4_C_H3NJCBGXK
Sample_RH4_DMSO_24h_H3K9me2_Rep3_C_HLTYHBGXM
Sample_RH4_DMSO_24h_H3K9me2_Rep5_C_H3NJCBGXK
Sample_RH4_PFI90_1uM_24h_H3K9me2_Rep3_C_HLTYHBGXM
Sample_RH4_PFI90_1uM_24h_H3K9me2_Rep5_C_H3NJCBGXK
Sample_RH4_DMSO_24h_PAX3FOXO1_Rep3_C_HWTJ5BGXK
Sample_RH4_DMSO_24h_PAX3FOXO1_Rep4_C_HWTJ5BGXK
Sample_RH4_PFI90_1uM_24h_PAX3FOXO1_Rep3_C_HWTJ5BGXK
Sample_RH4_PFI90_1uM_24h_PAX3FOXO1_Rep4_C_HWTJ5BGXK
Sample_RH4_DMSO_24h_HP1_Rep2_C_AAAWF2NM5
Sample_RH4_PFI90_1uM_24h_HP1_Rep2_C_AAAWF2NM5
Sample_RH4_DMSO_24h_KDM3B_C_HLTYHBGXM
Sample_RH4_PFI90_1uM_24h_KDM3B_C_HLTYHBGXM

Genome browser session
(e.g. UCSC)

Provide a link to an anonymized genome browser session for "Initial submission" and "Revised version" documents only, to enable peer review. Write "no longer applicable" for "Final submission" documents.

Methodology

Replicates

Replicates were performed for PAX3-FOXO1, H3K9me2, H3K27ac, H3K4me3 with n = 3. DiffBind was used to analyze the data with triplicates. The reproducibility was overall good between the replicates.

Sequencing depth

ChIP-seq was performed as single end reads with red length of 75 bp. Read depth ranged from 25 million - 54 million. Specific QC matrix for each sample is provided in Supplementary Table S7 Tab2.

Antibodies

PAX3-FOXO1, In House
H3K9me2, Abcam, ab1220
H3K4me3, Active motif, 39159
H3K27me3, Active motif, 39155
H3K27ac, Active motif, 39133
Total RNA Pol2, Millipore, 05-623
RNA Pol2 Ser5, Cell Signaling, 13523
RNA Pol2 Ser2, Cell Signaling, 13499
HP1, Cell Signaling, 2616
KDM3B, Cell Signaling, 3100

Peak calling parameters

ChIP-seq data was mapped to hg19 using BWA. We additionally mapped spike in reads to dm3 using BWA, and normalized human reads to million-mapped Drosophila reads (RRPM, reference normalize reads per million). Peaks were called using MACS2.0, with stringency thresholds of p = 0.0000001 and filtered to remove ENCODE blacklisted regions (satellite repeats and spuriously over-mapped regions).

Data quality

All QC matrix for each sample is provided on Supplementary Table S7 Tab2.

Software

Data was mapped to hg19 using BWA. For samples with spike in, we additionally mapped spike in reads to dm3 using BWA (version 0.7.17), and normalized human reads to million-mapped Drosophila reads (RRPM, reference normalize reads per million). Peaks were called using MACS2.0, with stringency thresholds of p = 0.0000001 and filtered to remove ENCODE blacklisted regions (satellite repeats and spuriously over-mapped regions). Codes used for ChIP-seq analyses is available on Github. https://github.com/CCRGeneticsBranch/khanlab_pipeline

Flow Cytometry

Plots

Confirm that:

- [x] The axis labels state the marker and fluorochrome used (e.g. CD4-FITC).
- [x] The axis scales are clearly visible. Include numbers along axes only for bottom left plot of group (a 'group' is an analysis of identical markers).
- [x] All plots are contour plots with outliers or pseudocolor plots.
- [x] A numerical value for number of cells or percentage (with statistics) is provided.

Methodology

Sample preparation

Trypsinized cultutred cells were washed, fixed and permeabilized using FOXP3/Perm Buffer (Biolegend, Cat. 421401)

Instrument	BD LSRFortessa™ Cell Analyzer
Software	FACSDiva software was used for data acquisition and FlowJo software was used for data analysis.
Cell population abundance	A minimum of 100k single cells were analyzed per sample. Relative cell abundance in each cell cycle stage was represented as a % of the total number of cycling cells (G0 + G1 + S + G2 + M)
Gating strategy	Cell debris and cell doublets were removed from analysis using FSC and SSC gates. Hoescht 3342 stain was used to determine cell 2n vs 4n ploidy cells. Specific stages of the cell cycle were determined as G0: 2n and Ki67- PH3-, G1: 2n and Ki67+ PH3-, S: >2n <4n and Ki67+ PH3-, G2: 4n and Ki67+ PH3-, M: 4n and Ki67+ PH3+.

☒ Tick this box to confirm that a figure exemplifying the gating strategy is provided in the Supplementary Information.
